# Body surface area estimation in children using weight alone: application in paediatric oncology

**DOI:** 10.1054/bjoc.2001.1859

**Published:** 2001-07

**Authors:** I Sharkey, A V Boddy, H Wallace, J Mycroft, R Hollis, S Picton

**Affiliations:** Pharmacy Dept, Royal Victoria Infirmary, Newcastle, UK; Cancer Research Unit, University of Newcastle, UK; Dept of Haematology and Oncology, Royal Hospital for Sick Children, Edinburgh, UK; Pharmacy Dept, Royal Marsden Hospital, London, UK; Paediatric Oncology and Haematology, St James University Hospital, Leeds, LS9 7TF, UK

## Abstract

The majority of chemotherapy regimens and trials specify doses of cytotoxic drugs normalized to body surface area. Estimation of BSA in paediatric patients is particularly problematic, as conventional nomograms require accurate determination of both height and weight. The chemotherapy standards group of the UKCCSG (United Kingdom Children's Cancer Study Group) has evaluated a method for calculation of body surface area (BSA) estimation, based solely on patient weight. In comparison with BSA estimations using 2 commonly used methods, which require both weight and height measurements, deviation in the estimate of BSA was less than 10%. This method may be extended to the dosing of chemotherapeutic agents in infants of body weight less than 10 kg, with appropriate recommendations for dose modification. Until better correlates of drug clearance, such as GFR for carboplatin, are identified BSA is used to standardize doses for most chemotherapeutic agents. The formula presented here provides a more robust and reliable method of calculation of BSA from weight alone. Although this approach has been shown to be equivalent to other currently used methods, care should be taken extending this calculation of BSA to children less than 10 kg, to obese patients and to those with cachexia. © 2001 Cancer Research Campaign http://www.bjcancer.com


British Journal of Cancer (2001) 85(1), 23–28
© 2001 Cancer Research Campaign
doi: 10.1054/ bjoc.2001.1859, available online at http://www.idealibrary.com on IDEAL®

### Body surface area estimation in children using weight alone: application in paediatric oncology

**I Sharkey[1], AV Boddy[2], H Wallace[3], J Mycroft[4], R Hollis[5] and S Picton[5]**
**on behalf of the Chemotherapy Standardisation group of the United Kingdom Children's Cancer Study Group**

[1]Pharmacy Dept, Royal Victoria Infirmary, Newcastle, UK; [2]Cancer Research Unit, University of Newcastle, UK; [3]Dept of Haematology and Oncology, Royal Hospital for Sick Children, Edinburgh, UK; [4]Pharmacy Dept, Royal Marsden Hospital, London, UK; [5]Paediatric Oncology and Haematology, St James University Hospital, Leeds, LS9 7TF, UK

**Summary** The majority of chemotherapy regimens and trials specify doses of cytotoxic drugs normalized to body surface area. Estimation of BSA in paediatric patients is particularly problematic, as conventional nomograms require accurate determination of both height and weight. The chemotherapy standards group of the UKCCSG (United Kingdom Children's Cancer Study Group) has evaluated a method for calculation of body surface area (BSA) estimation, based solely on patient weight. In comparison with BSA estimations using 2 commonly used methods, which require both weight and height measurements, deviation in the estimate of BSA was less than 10%. This method may be extended to the dosing of chemotherapeutic agents in infants of body weight less than 10 kg, with appropriate recommendations for dose modification. Until better correlates of drug clearance, such as GFR for carboplatin, are identified BSA is used to standardize doses for most chemotherapeutic agents. The formula presented here provides a more robust and reliable method of calculation of BSA from weight alone. Although this approach has been shown to be equivalent to other currently used methods, care should be taken extending this calculation of BSA to children less than 10 kg, to obese patients and to those with cachexia. © 2001 Cancer Research Campaign http://www.bjcancer.com

Drug disposition is critically dependent upon the physico-chemical characteristics of the drug itself, together with a number of physiological factors. In neonates, infants and children, those physiological factors which influence drug disposition (renal and hepatic function, metabolic rate) change rapidly during maturation (Crom et al, 1987, 1991; McLeod et al, 1992). These are accompanied by significant age-related changes in body composition (extracellular and total body water, fat distribution, lean body mass) (Friss-Hansen, 1961). The combined effect of these changes on the pharmacokinetic parameters of any drug may significantly influence systemic drug exposure. Drug clearance, and by definition systemic exposure, also shows wide inter- and intra-patient variation (Crom et al, 1987). This wide variation in systemic exposure may have a significant effect on disease response (Evans et al, 1998) and drug toxicity. Since changes in these physiological and pharmacokinetic parameters were thought to correlate most closely with Body Surface Area (BSA), it is this measurement which forms the basis of dose normalization with respect to variations in age, body size and body composition. This convention for drug dosing has recently been questioned (Gurney, 1996; Ratain, 1998), but aside from regimens incorporating pharmacokinetically guided treatment (Galpin and Evans, 1993; Desoize and Robert, 1994; Boos et al, 1995) and adaptive control of free-drug exposure, dosing of chemotherapeutic agents based on patient BSA is still the method employed in the majority of current paediatric treatment protocols.

*Received 18 October 2000*
*Revised 23 February 2001*
*Accepted 27 March 2001*

*Correspondence to:* S Picton

There are a large number of publications describing formulae and/or nomograms to estimate BSA from patient height and weight (DuBois and DuBois, 1916; Boyd, 1935; Sendroy and Cechini, 1952; Haycock and Schwarz, 1978; George and Gehan, 1979; Mosteller, 1987). Many of these formulae are derived by painstaking direct measurement of BSA using a variety of techniques, but often in a small number of subjects. The perceived advantages of any one of these methods in determining BSA in children, either in terms of accuracy or ease of use, are questionable and serious errors in the use of these techniques have also been described (Briars and Bailey, 1994). Similarly, sliding-scale BSA nomograms and BSA calculators produced by drug companies, and frequently used as 'aids to prescribing', are based almost universally on the formula described by Dubois (DuBois and DuBois, 1916). This formula was derived from a small subject sample ($n$ = 9), which included only one child and has been shown to significantly underestimate BSA in children less than 0.7 $m^2$ (Haycock and Schwarz, 1978).

Perhaps most importantly however, all of these methods require an accurate measurement of patient height and weight. Paediatricians and paediatric nurses will be familiar with the problems in obtaining accurate, reproducible measurement of patient height or supine length in children.

A method describing estimation of BSA from body weight (BW) alone has been described by Coulthard (Coulthard, 1994) and was based on one of the largest single bodies of work undertaken in direct surface-area measurement (Boyd, 1935). In the latter study, Boyd proposed that the application of a self-adjusting power equation (SAPE) in which weight is raised to a power which varies with its own value is so accurate in determining BSA that the 'advantage of using a similar equation requiring a height determinant was reduced to insignificance' (Coulthard, 1994).

**Table 1** BSA estimation in patients less than 10 kg. Values are calculated using the Boyd formula (1)

| Body weight (kg) | Surface area (m²) |
|---|---|
| 2 | 0.16 |
| 2.5 | 0.19 |
| 3 | 0.21 |
| 3.5 | 0.24 |
| 4 | 0.26 |
| 4.5 | 0.28 |
| 5 | 0.3 |
| 5.5 | 0.32 |
| 6 | 0.34 |
| 6.5 | 0.36 |
| 7 | 0.38 |
| 7.5 | 0.4 |
| 8 | 0.42 |
| 8.5 | 0.44 |
| 9 | 0.46 |
| 9.5 | 0.47 |
| 10 | 0.49 |

The application of the formula of Boyd to dosing in paediatric oncology regimens has been investigated in the current study. This evaluation led to the development of a simple table for use in paediatric oncology clinics for the dosing of drugs to children with cancer.

### METHODS

The analysis of Boyd was used to calculate BSA for patients over the range of weights typically seen in paediatric oncology (2–90 kg). Equation 1 gives the relationship between BSA (in $cm^2$) and weight (W) in g, as described by Boyd. This was corrected for the units of $m^2$ and kg in performing the analysis.

$$\mathrm{BSA} = 4.688 \bullet W^{(0.8168-0.0154 \cdot \log W)} \qquad (1)$$

Particular attention was paid to those patients weighing less than 10 kg (Table 1). Patients greater than 10 kg were examined separately (Table 2).

Since prescribers currently use a variety of methods to estimate BSA in their patients, this study has compared results obtained in a cohort of paediatric patients using the values in Tables 1 and 2 and two of the most commonly used alternative methods requiring a height and weight measurement:

(a) Mosteller formula:

$$\mathrm{BSA} = \sqrt{\frac{H \times W}{3600}} \qquad (2)$$

where BSA is in $m^2$, H (height) is in cm and W (weight) in kg.

(b) Sliding scale nomogram (pharmaceutical company prescribing aid), based on the DuBois formula:

$$A = W^{0.425} \times H^{0.725} \times 71.84 \qquad (3)$$

A = surface area in $cm^2$, H is height in cm and W is weight in kg.

Measurements of height and weight were carried out in the normal way for a cohort of patients ($n$ = 146) of both sexes, treated at 2 UKCCSG centres (100 from RVI, Newcastle and 46 from Birmingham Children's Hospital). The range of weights was 11.0 to 86.6 kg, with a median weight of 42.8 kg. None of the patients were clinically obese or cachectic. Children less than 10 kg were excluded as a reliable measure of height is not possible in these patients. BSA was estimated from Table 2 (Boyd formula) and compared with results obtained from the Mosteller formula and the DuBois nomogram. For each comparison, the percentage deviation of the Boyd formula from the conventional method was calculated. To determine if patients of differing sizes were more prone to bias or imprecision in BSA calculation, data were grouped into 3 weight categories: 10–30 kg, 31–50 kg and greater than 50 kg.

### RESULTS

When calculated using the Boyd formula (1), over the range of body weights considered for paediatric oncology patients, BSA varies from 0.16 to 0.49 $m^2$ for patients less than 10 kg (Table 1) and increases from 0.53 to 2.2 $m^2$ for patients weighing from 11 to 90 kg (Table 2). For all the data, the variations in BSA estimation between the Mosteller formula, the Dubois nomogram and the table based on the Boyd equation are displayed in Figures 1 and 2. The percentage variation of the Boyd formula from the estimates obtained by the algorithms requiring height is less than 15% in every case, and less than 10% in all but 2 patients in comparison with Mosteller, and 6 patients in comparison with Dubois. The proportions of patients within 5% of the estimate of BSA provided

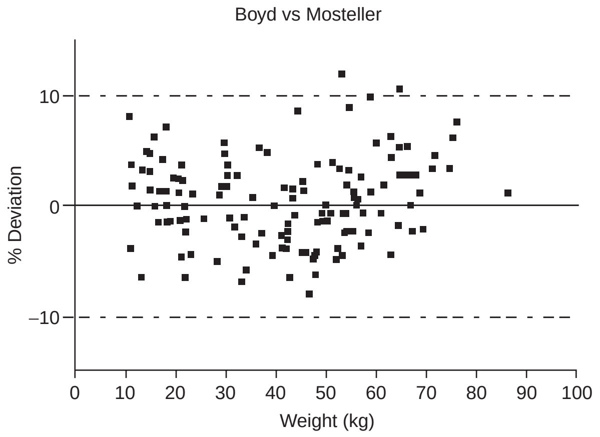

**Figure 1** Plot of the percentage deviation of the calculated surface area using the Boyd formula (1) based on weight alone, when compared to the Mosteller formula (2)

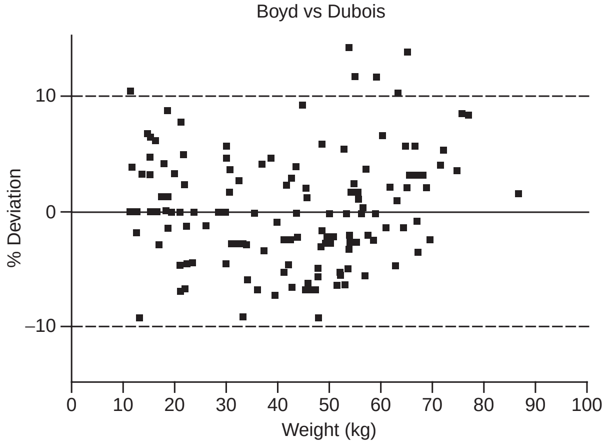

**Figure 2** Plot of the percentage deviation of the calculated surface area using the Boyd formula (1) based on weight alone, when compared to the Dubois formula (3)

© 2001 Cancer Research Campaign

**Table 2** BSA estimation in patients greater than 10 kg. Values are calculated using the Boyd formula (1)

| Body weight (kg) | Surface area ($m^2$) | Body weight (kg) | Surface area ($m^2$) |
|---|---|---|---|
| 11 | 0.53 | 51 | 1.5 |
| 12 | 0.56 | 52 | 1.5 |
| 13 | 0.59 | 53 | 1.5 |
| 14 | 0.62 | 54 | 1.6 |
| 15 | 0.65 | 55 | 1.6 |
| 16 | 0.68 | 56 | 1.6 |
| 17 | 0.71 | 57 | 1.6 |
| 18 | 0.74 | 58 | 1.6 |
| 19 | 0.77 | 59 | 1.7 |
| 20 | 0.79 | 60 | 1.7 |
| 21 | 0.82 | 61 | 1.7 |
| 22 | 0.85 | 62 | 1.7 |
| 23 | 0.87 | 63 | 1.7 |
| 24 | 0.9 | 64 | 1.7 |
| 25 | 0.92 | 65 | 1.8 |
| 26 | 0.95 | 66 | 1.8 |
| 27 | 0.97 | 67 | 1.8 |
| 28 | 1.0 | 68 | 1.8 |
| 29 | 1.0 | 69 | 1.8 |
| 30 | 1.1 | 70 | 1.9 |
| 31 | 1.1 | 71 | 1.9 |
| 32 | 1.1 | 72 | 1.9 |
| 33 | 1.1 | 73 | 1.9 |
| 34 | 1.1 | 74 | 1.9 |
| 35 | 1.2 | 75 | 1.9 |
| 36 | 1.2 | 76 | 2.0 |
| 37 | 1.2 | 77 | 2.0 |
| 38 | 1.2 | 78 | 2.0 |
| 39 | 1.3 | 79 | 2.0 |
| 40 | 1.3 | 80 | 2.0 |
| 41 | 1.3 | 81 | 2.0 |
| 42 | 1.3 | 82 | 2.1 |
| 43 | 1.3 | 83 | 2.1 |
| 44 | 1.4 | 84 | 2.1 |
| 45 | 1.4 | 85 | 2.1 |
| 46 | 1.4 | 86 | 2.1 |
| 47 | 1.4 | 87 | 2.1 |
| 48 | 1.4 | 88 | 2.2 |
| 49 | 1.5 | 89 | 2.2 |
| 50 | 1.5 | 90 | 2.2 |

by the more conventional methods were 84% and 73% respectively. There was no effect of gender on the concurrence of the different calculation methods.

Categorization of the subjects according to weight did not reveal any trends to under- or overestimation of BSA using the Boyd equation compared to Mosteller or Dubois. Mean prediction error (MPE), or bias, was 1.1, –1.8 and 2.1% for patients less than 30 kg, 30–50 kg and greater than 50 kg respectively (comparing Boyd with Dubois). Corresponding values for mean absolute prediction error (MAPE), or precision, were 3.3, 3.8 and 4.2%. Comparison of Boyd with Mosteller produced values for MPE and MAPE indicating less bias and higher precision in each of the weight categories.

Reliable data were available only from patients weighing over 10 kg. To assess the impact of implementing the Boyd formula in smaller patients, calculations were made of the dosing implications for a typical child on a paediatric drug treatment protocol (Table 3). In this protocol, drugs are administered at doses based on BSA for patients greater than 10 kg, and based on weight for smaller children. Dosing based on surface area would result in an increase in dose for children less than 10 kg of between 65 and 92%, compared to current practise, in this protocol.

### DISCUSSION

Dosing based on BSA is founded largely in the history of cytotoxic drug development (Ratain, 1998) and despite its many limitations remains the method employed in the majority of current treatment protocols. With the majority of cytotoxic agents, drug clearance and by definition systemic exposure correlates only loosely with either body weight or BSA. In particular, there is little sound physiological, pharmacokinetic or pharmacodynamic data to support BSA-based dosing of many cytotoxic drugs. In some cases, closer correlations exist between some other measurable parameter e.g. carboplatin clearance and glomerular filtration rate (GFR) and this has formed the basis for GFR-based dosing regimes in many paediatric and adult treatment protocols (Calvert et al, 1989; Newell et al, 1993). These variations in inter-individual drug handling have stimulated recent research into the individualization of treatment of paediatric cancers in order to optimize outcome

*© 2001 Cancer Research Campaign* 

**Table 3** Comparison of chemotherapy dosing using body weight v body surface area

| Drug | Dose by weight | Dose by SA | Ratio of doses |
|---|---|---|---|
| Vincristine | | | |
| 1.5 mg $m^{-2}$<br>0.05 mg $kg^{-1}$ | 0.25 mg | 0.45 mg | 1.80 |
| Carboplatin | | | |
| 550 mg $m^{-2}$<br>20 mg $kg^{-1}$ | 100 mg | 165 mg | 1.65 |
| Methotrexate | | | |
| 8 g $m^{-2}$<br>250 mg $kg^{-1}$ | 1.25 g | 2.4 g | 1.92 |
| Cisplatin | | | |
| 40 mg $m^{-2}$<br>1.3 mg $kg^{-1}$ | 6.5 mg | 12 mg | 1.85 |

Example: Protocol UKCCSG Skudy CNS 9204 (Baby Brain). Patient wt = 5 kg.
Estimated BSA = 0.3 $m^2$ (from Table 1) using the Boyd formula.

and reduce drug toxicity (Evans et al, 1998). However, with the exceptions of carboplatin, methotrexate and 6-mercaptopurine, no rational approach to individualized dosing of drugs administered to paediatric patients has emerged, and surface area is the most commonly used parameter with which to adjust doses for the wide range of body sizes encountered in paediatric oncology.

Many methods have been employed and formulae derived to estimate BSA. These methods should not be accepted as a precise measurement of BSA, but rather as techniques which will allow comparison between individuals (Pinkel, 1958). Against this background, we have identified and validated a method which standardizes BSA measurement in children and reduces the possible errors attendant in the use of nomograms and formulae. Given the difficulties in measuring height in children and the implications that this may have on the accuracy of any calculation, the table derived from the Boyd formula estimates BSA values without significant variation from either of the 2 methods tested in this study. These comparisons are not absolute, as there is no definitive estimate of BSA offered by any of the proposed methods. However, the method proposed here is simpler and easier to apply in a more consistent manner. This should reduce errors in dose calculation and provide more uniformity of doses administered, thus removing possible confounding effects in the interpretation of multicentre clinical trials.

This method has been validated in children greater than 10 kg, and can be recommended for application to drug protocols for this group of patients. However, the majority of paediatric protocols apply a cut-off for surface area based dosing at 10 kg. Below this weight, the dosing of chemotherapeutic drugs is specified on a mg $kg^{-1}$ basis, usually based on an approximate extrapolation from a 1 $m^2$ individual assumed to weight 30 kg, and assuming a linear relationship between weight and BSA. However, in children less than 10 kg body weight, this results in a significant reduction in dose compared to the reference dose for a larger child calculated on the basis of BSA (Table 3).

This cautious approach to drug dosing in infants and younger children is designed to avoid excessive myelosuppression and other significant drug toxicities resulting from impaired drug elimination (reduced biliary excretion, decreased renal-tubular excretion, hepatic enzyme immaturity) in very young infants (< 6 kg) (Woods et al, 1981; Reaman, 1993).

However, the assumption of reduced hepatic and renal function has not been borne out by recent investigations (Newell et al, 1993; Blanco et al, 2000) and these adjustments are applied across a significant weight range. There is a likelihood that sub-therapeutic dosing occurs in some patients within this group. Also there is the problem of the significant step-up in dosage as children cross the 10 kg boundary, and move from mg $kg^{-1}$ to mg $m^{-2}$ dosing during their treatment. Both of these issues have implications in terms of both disease response and drug toxicity.

In the context of the clinical application of the Boyd formula, the UKCCSG has recommended that the BSA table be used to estimate body surface area in infants under 10 kg in weight in order to provide a smoother transition in dosing for this group of patients. It is recommended that the question of dose reduction in infants less than 12 months of age should be addressed by individual investigators and protocols. In order to assist protocol designers in the selection of appropriate starting doses of chemotherapeutic agents in children less than 12 months of age or less than 10 kg body-weight, guidelines have been produced by the UKCCSG and are attached as an appendix.

There are limits to the application of any algorithmic method in the calculation of drug dosages (Gurney, 1996; Smith, 1996). In severely malnourished or obese patients BSA estimation based on any algorithm with a weight parameter may result in inappropriate dosing. The tables proposed here for the estimation of BSA, and thus cytotoxic drug doses, must be taken in conjunction with a clinical assessment of the patient, including the implications of co-existing illness, previous chemo- or radiotherapy, concurrent drug treatment and nutritional status.

Concern has been expressed in a number of publications and editorials that paediatric patients may be receiving inappropriate doses because of inadequate methods for the estimation of BSA, and lack of standardization of methods between study centres. To address this concern, and to provide standardized dosing of chemotherapeutic agents in UKCCSG trials, the Boyd estimates of BSA, based on body weight alone, have been used to construct a table from which the BSA can be calculated. The table is simple and uncomplicated to use, and overcomes the significant errors in determining height measurements in children and infants.

### ACKNOWLEDGEMENTS

This work was supported by the Cancer Research Campaign.

 © 2001 Cancer Research Campaign

### REFERENCES

Blanco JG, Harrison PL, Evans WE and Relling MV (2000) Human cytochrome P450 maximal activities in pediatric versus adult liver. *Drug Metab Disp* **28**: 379–382

Boos J, Krumpelmann S, Sculze-Westhoff P, Euting T, Berthold F and Jurgens H (1995) Steady state levels and bone marrow toxicity of etoposide in children and infants. Does etoposide require age-dependent dose calculation. *J Clin Oncol* **13**: 2954–2960

Boyd E (1935) *Surface Area of the Human Body*. University of Minnesota Press: Mineapolis

Briars GL and Bailey BJR (1994) Surface area estimation: Pocket calculator v nomogram. *Archives of Disease in Childhood* **70**: 246–247

Calvert AH, Newell DR, Gumbrell LA, O'Reilly S, Burnell M, Boxall FE, Siddik ZH, Judson IR, Gore ME and Wiltshaw E (1989) Carboplatin dosage: prospective evaluation of a simple formula based on renal function. *J Clin Oncol* **7**: 1748–1756

Coulthard MG (1994) Surface area is best estimated from weight alone: Pocket calculators and nomograms are unnecessary. *Archives of Disease in Childhood* **71**: 281

Crom WR, Glynn-Barnhart AM, Rodman JH, Teresi ME, Kavanagh RK, Christensen ML, Relling MV and Evans WE (1987) Pharmacokinetics of anticancer drugs in children. *Clin Pharmacokin* **12**: 168–213

Crom WR, Relling MV, Christensen ML, Rivera GK and Evans WE (1991) Age-related differences in hepatic drug clearance in children: Studies with lorazepam and antipyrine. *Clin Pharmacol Ther* **50**: 132–140

Desoize B and Robert J (1994) Individual dose adaptation of anticancer drugs. *Eur J Cancer* **30A**: 844–851

DuBois D and DuBois EF (1916) A formula to estimate the approximate surface area if height and weight be known. *Archives of Internal Medicine* **17**: 863–871

Evans WE, Relling MV, Rodman JH, Crom WR, Boyett JM and Ching-Hon P (1998) Conventional compared with individualised chemotherapy for childhood acute lymphoblastic leukemia. *N Eng J Med* **338**: 499–505

Friss-Hansen B (1961) Body water compartments in children; changes during growth and related changes in body composition. *Pediatrics* **28**: 169–181

Galpin A and Evans W (1993) Therapeutic drug monitoring in cancer management. *Clin Chem* **39**: 2419–2430

George SL and Gehan EA (1979) Methods for measurement of body surface area. *Journal of Pediatrics* **94**: 342–343

Gurney H (1996) Dose calculation of anticancer drugs: a review of the current practice and introduction of an alternative. *J Clin Oncol* **14**: 2590–2611

Haycock GB and Schwarz GJ (1978) Geometric method for measuring body surface area. A height-weight formula validated in infants. *J Paed* **93**: 62–66

McLeod HL, Relling MV, Crom WR, Silverstein K, Groom S, Rodman JH, Rivera GK, Crist WM and Evans WE (1992) Disposition of antineoplastic agents in the very young child. *Br J Cancer* **66**: S 23–S 29

Mosteller RD (1987) Simplified calculation of body surface area. *N Eng J Med* **317**: 1098

Newell DR, Pearson ADJ, Balmanno K, Price L, Wyllie RA, Kier M, Calvert AH, Lewis IJ, Pinkerton CR and Stevens MCG (1993) Carboplatin pharmacokinetics in children: the development of a pediatric dosing formula. *J Clin Oncol* **11**: 2314–2323

Pinkel D (1958) The use of body surface area as a criterion of drug dosage in cancer chemotherapy. *Cancer Res* **18**: 853

Ratain MJ (1998) Body-surface area as a basis for dosing of anticancer agents. *J Clin Oncol* **16**: 2297–2298

Reaman GH (1993) Special consideration for the infant with cancer. In *Principles and Practise of Paediatric Oncology* Pizzo PA and Poplack DG (eds) pp. 303–314. J.B. Lippincott Company: Philadelphia, Pa

Sendroy J and Cechini LP (1952) Determination of human body surface area from height and weight. *Journal of Applied Physiology* **7**: 1–12

Smith JM (1996) Dangers of algorithms. *Pharmaceutical Journal* **257**: 136

Woods WG, O'Leary M and Nesbitt ME (1981) Life-threatening neuropathy and hepatotoxicity in infants during induction therapy for acute lymphoblastic leukemia. *Journal of Pediatrics* **98**: 642–645

*© 2001 Cancer Research Campaign*

**Appendix** Guidelines for dose adjustments in children less than 10 kg or less than 12 months of age

**Caution**

- For children less than 10 kg body-weight, dosing by body surface area represents a change in usual clinical practice: This will result in an **increase** in calculated dose.
- The implications of this change, in clinical practice, are not known in terms of drug toxicity.
- Recommendations:

**Starting doses:** For infants **less than 6 months** of age:
**50%** of calculated dose by body surface area.
For infants **6 months to 1 year** of age:
**75%** of calculated dose by body surface area.
For infants **over 1 year** of age:
**100%** of calculated dose by body surface area.

- These doses may be adjusted according to clinical circumstances.
- Individual investigators (and protocols) should have **clear** recommendations for dosing in infants, and should monitor both disease response and toxicity closely in order to identify any clinical problems related to change in chemotherapy doses.

*© 2001 Cancer Research Campaign*

## References

[PDF_00439] Blanco J. G., Harrison P. L., Evans W. E., Relling M. V. (2000). Human cytochrome P450 maximal activities in pediatric versus adult liver.. Drug Metab Dispos.

[PDF_00442] Boos J., Krümpelmann S., Schulze-Westhoff P., Euting T., Berthold F., Jürgens H. (1995). Steady-state levels and bone marrow toxicity of etoposide in children and infants: does etoposide require age-dependent dose calculation?. J Clin Oncol.

[PDF_00450] Calvert A. H., Newell D. R., Gumbrell L. A., O'Reilly S., Burnell M., Boxall F. E., Siddik Z. H., Judson I. R., Gore M. E., Wiltshaw E. (1989). Carboplatin dosage: prospective evaluation of a simple formula based on renal function.. J Clin Oncol.

[PDF_00454] Coulthard M. G. (1994). Surface area is best estimated from weight alone: pocket calculators and nomograms are unnecessary.. Arch Dis Child.

[PDF_00457] Crom W. R., Glynn-Barnhart A. M., Rodman J. H., Teresi M. E., Kavanagh R. E., Christensen M. L., Relling M. V., Evans W. E. (1987). Pharmacokinetics of anticancer drugs in children.. Clin Pharmacokinet.

[PDF_00460] Crom W. R., Relling M. V., Christensen M. L., Rivera G. K., Evans W. E. (1991). Age-related differences in hepatic drug clearance in children: studies with lorazepam and antipyrine.. Clin Pharmacol Ther.

[PDF_00463] Desoize B., Robert J. (1994). Individual dose adaptation of anticancer drugs.. Eur J Cancer.

[PDF_00467] Evans W. E., Relling M. V., Rodman J. H., Crom W. R., Boyett J. M., Pui C. H. (1998). Conventional compared with individualized chemotherapy for childhood acute lymphoblastic leukemia.. N Engl J Med.

[PDF_00470] FRIIS-HANSEN B. (1961). Body water compartments in children: changes during growth and related changes in body composition.. Pediatrics.

[PDF_00472] Galpin A. J., Evans W. E. (1993). Therapeutic drug monitoring in cancer management.. Clin Chem.

[PDF_00474] George S. L., Gehan E. A. (1979). Methods for measurement of body surface area.. J Pediatr.

[PDF_00476] Gurney H. (1996). Dose calculation of anticancer drugs: a review of the current practice and introduction of an alternative.. J Clin Oncol.

[PDF_00478] Haycock G. B., Schwartz G. J., Wisotsky D. H. (1978). Geometric method for measuring body surface area: a height-weight formula validated in infants, children, and adults.. J Pediatr.

[PDF_00485] Newell D. R., Pearson A. D., Balmanno K., Price L., Wyllie R. A., Keir M., Calvert A. H., Lewis I. J., Pinkerton C. R., Stevens M. C. (1993). Carboplatin pharmacokinetics in children: the development of a pediatric dosing formula. The United Kingdom Children's Cancer Study Group.. J Clin Oncol.

[PDF_00489] PINKEL D. (1958). The use of body surface area as a criterion of drug dosage in cancer chemotherapy.. Cancer Res.

[PDF_00491] Ratain M. J. (1998). Body-surface area as a basis for dosing of anticancer agents: science, myth, or habit?. J Clin Oncol.

[PDF_00496] SENDROY J., CECCHINI L. P. (1954). Determination of human body surface area from height and weight.. J Appl Physiol.

[PDF_00500] Woods W. G., O'Leary M., Nesbit M. E. (1981). Life-threatening neuropathy and hepatotoxicity in infants during induction therapy for acute lymphoblastic leukemia.. J Pediatr.

